# Identifying Issues and Priorities in Reporting Back Environmental Health Data

**DOI:** 10.3390/ijerph17186742

**Published:** 2020-09-16

**Authors:** Erin Lebow-Skelley, Sarah Yelton, Brandi Janssen, Esther Erdei, Melanie A. Pearson

**Affiliations:** 1 HERCULES Exposome Research Center, Rollins School of Public Health, Emory University, Atlanta, GA 30322, USA; mapears@emory.edu; 2 Institute for the Environment, UNC Superfund Research Program, University of North Carolina at Chapel Hill, Chapel Hill, NC 27599, USA; sarah.yelton@unc.edu; 3 Department of Occupational and Environmental Health, University of Iowa, Iowa City, IA 52246, USA; brandi-janssen@uiowa.edu; 4 College of Pharmacy & Mountain West Clinical and Translational Research-Infrastructure Network, UNM Health Sciences Center, University of New Mexico, Albuquerque, NM 87131, USA; EErdei@salud.unm.edu

**Keywords:** concept mapping, research report-back, environmental health, community engagement

## Abstract

Experts recommend reporting environmental exposure results back to research participants and communities, yet environmental health researchers need further guidance to improve the practice of reporting back. We present the results of a workshop developed to identify pertinent issues and areas for action in reporting back environmental health research results. Thirty-five attendees participated, brainstorming responses to the prompt: “What are some specific issues that are relevant to reporting back research results to individuals or the larger community?”, and then grouping responses by similarity and rating their importance. Based on a combined theoretical foundation of grounded theory and qualitative content analysis, we used concept mapping to develop a collective understanding of the issues. Visual maps of the participants’ responses were created using nonmetric multidimensional scaling and hierarchical cluster analysis. The resulting concept map provided a spatial depiction of five issue areas: Effective Communication Strategies, Community Knowledge and Concerns, Uncertainty, Empowering Action, and Institutional Review and Oversight (listed from highest to lowest rating). Through these efforts, we disentangled the complex issues affecting how and whether environmental health research results are reported back to participants and communities, by identifying five distinct themes to guide recommendations and action. Engaging community partners in the process of reporting back emerged as a unifying global theme, which could improve how researchers report back research results by understanding community context to develop effective communication methods and address uncertainty, the ability to act, and institutional concerns about beneficence and justice.

## 1. Introduction

Environmental health research often involves measuring levels of chemicals in human fluids (e.g., blood, saliva, and urine) and/or in environmental samples (e.g., soil, water, air, food, and household dust). Over the last 15 years, considerable discussion among environmental health researchers has focused on whether or not and how this type of exposure data should be reported back to research participants and/or the affected community. Much of the debate included considerations of ethical issues [1], utility [2], and trust between researchers and research participants [3]. While many of these considerations are still relevant, a recent National Academies of Sciences, Engineering, and Medicine [4] report recommends routine return of results to research participants, primarily because “of the larger cultural transition toward more engagement, collaboration, and transparency between investigators and research participants” (page ix, preface). Similarly, the Consortium to Perform Human biomonitoring on a European Scale also recommended reporting personal results to participants [5]. After in-depth ethical discussions, investigators in a Canadian-based national biomonitoring initiative developed a decision-tree for reporting biomonitoring results to participants based on available health-based guidelines and population reference ranges [6].

Reporting back can refer to the return of research results to an individual research participant and/or a larger community. Within the context of environmental health, data reported back to an individual may include contaminant levels measured in a research participant’s bodily fluids/tissues or in environmental media collected in the research participant’s immediate environment (e.g., indoor dust samples from the participant’s home or ambient air samples from a participant’s yard). In contrast, reporting back at the community-level focuses on the aggregate results of a study. Both types of reporting back will often provide a comparison to help research participants contextualize their personal exposure or the community-level risk. Paper or digital report is a very common format for reporting back, however, exposure results may also be reported back in-person, either individually or in a community meeting [7,8]. Despite the many recommendations to return research results to study participants, there continue to be many barriers to implementing this practice, including financial support, lack of expertise, lack of established approaches, and institutional approvals [9].

In light of the increased attention to this topic and the challenges it presents, the organizers of the 2018 National Institute of Environmental Health Science’s (NIEHS) Partnerships for Environmental Public Health (PEPH) Annual Meeting selected reporting back research results as the meeting theme. For two days, meeting participants discussed challenges and shared approaches for report-back [10]. The NIEHS PEPH is a network of scientists, community members, educators, healthcare providers, public health officials, and policymakers, many of whom are NIEHS grantees who collaborate on environmental health research [11]. Many PEPH members are affiliated with a Community Engagement Core that is part of an NIEHS-funded Center (such as the Environmental Health Sciences Core Centers or Superfund Research Programs) [12]. Community Engagement Core teams, which often include social and/or behavioral scientists, translate research findings from their center into information for affected communities, the general public, decision makers, and health care professionals [13]. As such, they have become increasingly interested in the importance of and considerations around reporting back research results [10].

In an effort to further explore these considerations and develop recommendations to improve the practice of reporting back, we conducted a workshop with members of the PEPH community to identify issues relevant to reporting back within the context of environmental health research. Using a collaborative approach and building from the perspectives and experiences of a subset of the PEPH community, we aimed to (1) identify themes relevant to reporting back, (2) determine the relative importance of each theme, (3) understand connections between themes, and (4) organize the information into a useful framework to develop recommendations for reporting back research results. The foundation for this approach is a mix of grounded theory and qualitative content analysis. Grounded theory has taken different forms since it was originally defined by Glaser and Strauss [14], and our application most closely reflects Charmaz’s definition of grounded theory as a method of research that explores social processes (such as the process of reporting back) and creates conceptual frameworks from the data [15]. In our approach, similar to grounded theory, the perspectives of those providing the data are included in the interpretation of the data [16]. There are also several definitions of qualitative content analysis, with our approach aligning most closely with Roller and Lavraka’s [17] definition of a systematic reduction of content to identify themes and extract meaningful interpretations of the data. Similar to content analysis [17], our approach included a systemic process to extract themes and underlying meanings of these themes from the data. Lastly, our efforts to identify and describe relationships among the themes and the acceptance of our interpretive roles as researchers also reflects a grounded theory approach [16,18].

To achieve our aims, we used a concept mapping method originally proposed by Trochim and Linton [19], which shares characteristics with both grounded theory and content analysis. This structured method allows a group to identify their ideas and represent them visually. Concept mapping has its roots in ethnographic methods that elicit cultural domains of knowledge. Cultural domains are categories of knowledge held in common by particular social groups and can be most simply defined as “a set of items all of which a group of people define as belonging to the same type” [20]. Importantly, parts of the concept mapping process are specifically designed to elicit knowledge without interference from the researcher [21]. Concept mapping is a well-documented participatory mixed methods approach (qualitative and quantitative), which has been used internationally for conceptualizing themes from participant input, building meaning among themes, and constructing a conceptual framework [22].

Concept mapping approaches are often applied to assist groups in planning and evaluation [19,23]. An important component of the concept mapping process is that the resulting concept maps and their interpretation should be utilized for clarifying perspectives, setting priorities, stimulating new ideas, or planning for action. The participants in our concept mapping workshop chose to utilize their group conceptualization by further exploring the issues that emerged and, in line with the subject at hand, reporting their conceptualization back to the environmental health community. As such, this report summarizes the use of concept mapping to disentangle the myriad issues associated with reporting back environmental health results to participants and communities. Building from the conceptualization provided by practitioners, we summarize the emerging themes within the context of the relative importance of each theme and the interconnections among them. Then, from this conceptual framework, we make recommendations for improving the practice of reporting back environmental health data, which has the potential to improve environmental health education and communication, health behaviors, and overall public health.

## 2. Materials and Methods

The Community Engagement Core from Emory University’s HERCULES Exposome Research Center developed the 2018 PEPH concept mapping workshop following the concept mapping steps defined by Trochim [23], which are outlined in Table 1. Table 1 also indicates how this process was operationalized with the PEPH community, which is described in more detail below. The Emory Institutional Review Board (IRB) reviewed the workshop protocol and determined that it did not meet the definition of research with human subjects.

### 2.1. Step 1: Preparation

The HERCULES staff using Group Concept Mapping at the annual meeting to elicit and identify issues and recommended actions around the annual meeting’s topic of reporting back research results. The workshop, titled “Group Concept Mapping: Transforming individual experiences into community knowledge for action” was included as one of four concurrent workshop options for attendees to select as part of the Annual Meeting registration. An overview of the participants is provided below.

In collaboration with NIEHS PEPH staff, the following focus and rating prompts were developed to create a collective understanding of the issues and priorities these community-engaged researchers and practitioners have regarding reporting back:

**Focus prompt:**  “*What are some specific issues that are relevant to reporting back research results to individuals or the larger community*?”

**Rating prompt:**  “*How important is this issue to successful report-back*?”

**Participant Overview:** One-hundred and forty-three people registered to attend the 2018 PEPH Annual Meeting, representing academic institutions (66%), government (primarily NIEHS, 22%), community organizations (11%), and local public health (1%). Thirty-five attendees chose to participate in the concept mapping workshop, representing academic institutions (63%), NIEHS (11%), community organizations (23%), and local public health (3%). Overall, workshop attendees reflected the meeting registration, with a smaller proportion of government representatives and higher proportion of community representatives. All participants were affiliated with an NIEHS-funded research center or research project with a community-engaged-research component, as either the funder, academic researcher, community-engagement core staff, or community partner. The majority (n = 21) were Community Engagement Core staff, described in the introduction. All participants contributed to brainstorming, 26 completed sorting, and 21 completed rating (some attendees paired up to complete the online components, while others chose not to participate). Three workshop participants, in addition to the two HERCULES staff members, participated in the additional interpretation and utilization steps reported here.

### 2.2. Step 2: Generation of Statements

In order to generate a list of statements, we used a modified nominal group technique [24], in which participants each wrote three responses to the focus prompt (issues relevant to reporting back) on a piece of paper, and then each shared one answer out loud until all ideas were provided.

### 2.3. Step 3: Structuring of Statements

During the workshop, HERCULES staff uploaded the completed list of statements into The Concept System^®^ Global MAX™ software (Concept Systems, Inc., Ithaca, USA, Copyright 2004–2020; all rights reserved) and provided participants with an online invitation link to individually sort each statement into groups of statements that they thought were conceptually related. Participants then labeled each of their piles with a name that made sense to them. Lastly, participants were asked to individually rate each statement on a scale of 1–5 (1 = not at all important, 5 = absolutely essential): “How important is this issue to successful report-back?”

### 2.4. Step 4 and 5: Representation of Statements and Interpretation of Maps

**Figure 1:**
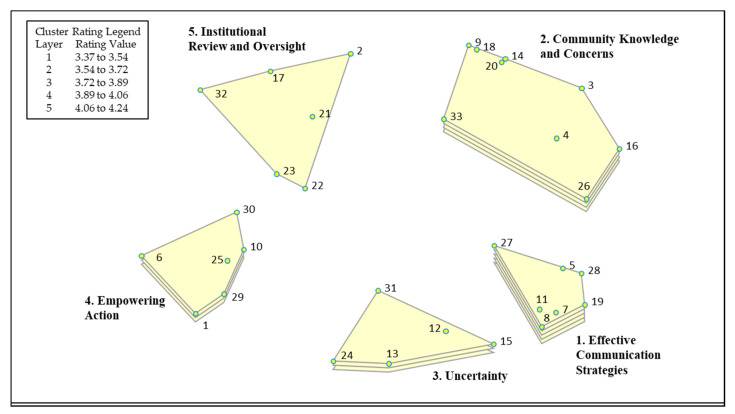
Report-back Cluster Rating Map. The Cluster Rating Map depicts the five report-back themes identified and selected by workshop participants, including points and statement numbers for each statement that makes up the cluster (listed in Table 1). Statements that were frequently sorted together (seen by participants as conceptually related) are placed closer to each other. The average importance rating of each cluster is illustrated by the layers of each cluster, with more layers indicating a higher average rating.

The Concept System^®^ Global MAX™ software creates visual representations of statements by first locating each statement as a separate point on a two-dimensional map using nonmetric multidimensional scaling [23]. On the point map, statements that participants frequently sorted together are closer to each other. For example, in Figure 1, which includes the point map that resulted from the nonmetric multidimensional scaling of our participants’ sorting data, statements 20 (“Undervaluing community knowledge”) and 14 (“Bias against community members from academics”) are adjacent to each other, meaning participants frequently sorted these issues together (i.e., saw them as related). The software then groups those statements, or points, into clusters, using hierarchical cluster analysis. Each cluster represents a distinct theme that emerged from the participants’ sorting data (i.e., how each individual sorted the brainstormed statements). Like the points on the map, the clusters that are closer to each other are more closely related conceptually, and vice versa.

The software produces multiple maps, representing different variations of clusters that represent distinct themes. The software also provides possible labels for these clusters using the labels provided during the sorting process by participants whose clusters most closely reflect the final map.

Determining the final concept map is inherently subjective, yet participatory. HERCULES staff presented the six cluster variations calculated by the software to the workshop participants, with maps that ranged from five to ten distinct themes. As part of a group discussion, participants gave initial impressions on the different cluster variations and cluster labels, selecting a map with five themes that they felt best represented their perspective (for example, the participants saw two clusters that both described issues related to community and decided to choose a map in which those clusters were combined into one larger community theme). After the workshop, all participants were sent a survey and invited to a conference call to confirm the final concept map and cluster labels (theme names) and discuss utilization of the map. This group (*n* = 9) confirmed the final map depicting five distinct themes relevant to reporting back.

The software also provides rating and bridging data for the concept map, which allows users to interpret the collective priorities of the participants and the cohesiveness of the identified themes. Rating values are provided for individual statements and as average values for each cluster. Bridging values reflect cohesiveness of the theme, with a higher bridging score indicating a theme with many statements that “bridged” to other areas of the map and, thus, is a less coherent theme and more interrelated with the other themes.

### 2.5. Step 6: Utilization of Maps

The majority of workshop participants indicated that the information illustrated by the concept maps was useful for the environmental health community and recommended that it should be shared with a wider audience. A subgroup of workshop participants from different academic institutions volunteered to proceed with map utilization (the co-authors of this manuscript). Consistent with the grounded theory approach [16], the subgroup further interpreted the map results to expand on each theme and develop theme-specific recommendations. The authors incorporated the rating and bridging data into their interpretation to identify priorities regarding report back and the inter-relationships between themes. For example, the issues seen as the most important to successful report back may help to set priorities for further action, and those issues that are most pertinent to their theme (the anchors with low bridging) help to define the interpretation of that theme. Meanwhile, issues that bridge to other themes indicate that recommended actions may be interrelated. This manuscript presents that further interpretation of the 2018 PEPH Report-back Concept Map in hopes that the wider environmental health community may utilize the results and recommendations to improve the practice of reporting back of environmental health data.

## 3. Results

### 3.1. Cluster Map

**Figure 2:**
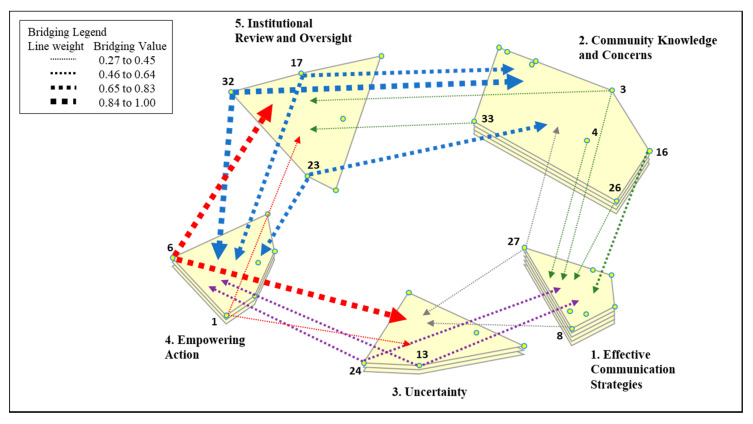
Cluster Rating Map with Highly Bridged Statements. Arrows come from statements with bridging values above the cluster mean and point towards the cluster that they were frequently sorted with. The weight of the arrow indicates the bridging value, with heavier lines indicating a statement that was more frequently sorted with other clusters, i.e., spanned to other areas of the map because participants conceptualized them as interrelated with other themes.

The final map includes five clusters, representing the following themes: Effective Communication Strategies, Community Knowledge and Concerns, Uncertainty, Empowering Action, and Institutional Review and Oversight (Figure 1 and Table 2). The final stress value for the point map was 0.23, which is average for concept mapping projects and indicates goodness of fit [25]. Each statement that forms a cluster is listed in Table 2, along with its rating and bridging values, demonstrating the specific issues that make up each report-back theme and their relative importance and interrelatedness. Individual statement ratings ranged from 2.85 to 4.95, and the average cluster rating ranged from 3.37 to 4.24 (mean 3.84). While the ratings varied, it should be noted that each cluster’s emergence as a discreet theme reflects that it was identified as an issue relevant to reporting back. Average cluster bridging ranged from 0.13 to 0.62 (mean 0.36), with individual statements ranging from 0.27 to 1.00. Figure 2 illustrates the relationships between clusters (or themes) by depicting the statements that had bridging values above their cluster mean, with arrows from those statements pointing towards the clusters they were frequently sorted with. These arrows help to illustrate the significance of the placement of the clusters on the map. For example, Cluster 5, Institutional Review and Oversight, had three statements with relatively high bridging values above the cluster mean (32, 17, 23). Participants sorted these statements frequently with statements in the Empowering Action and Community Knowledge and Concerns clusters, indicating that this was a less conceptually cohesive cluster and that participants thought these issues were related to issues regarding community knowledge and empowering action. In this way, Group Concept Mapping visualizes distinct conceptual clusters of ideas while also calculating and visualizing the complexity and interrelationships among concepts. We describe each theme’s results below, including its relative importance (cluster rating), cohesiveness and interrelationships (bridging), and the identification of prominent subthemes. We then use the importance ratings, interrelationships, and the existing literature, to further interpret the themes and their utility for action in the Discussion section.

### 3.2. Clusters

#### 3.2.1. Cluster One: Effective Communication Strategies

The communication theme reflects the need to use effective communication approaches when sharing report-back information and messages to participants. This theme received the highest rating (4.24), suggesting that the participating members of the PEPH community found issues around effective communication to be most important to successfully reporting back information to participants. This theme also had the lowest average bridging (0.13) among all the clusters, meaning participants conceptualized issues around communication as a cohesive and coherent theme. The most highly rated issues, as well as those most pertinent to the theme (with the lowest bridging values), involved the practical considerations of communicating information to study participants to ensure they understand what it means: knowing “what language to deliver it in”, “what medium to use/how to deliver it”, “defining scientific measurement/terms”, and “how to represent it visually” (Table 2, Statements 28, 19, 11, 7).

Within this theme, the two issues with the highest bridging values were related to the “Uncertainty” theme, in particular with issues related to determining what information to share in report-back materials and how to communicate about uncertainty (see Figure 2 and Table 2).

#### 3.2.2. Cluster Two: Community Knowledge and Concerns

This theme reflects issues with reporting back that are specific to involving communities. These issues revolve around including and valuing community knowledge in the process of reporting back and the challenges in doing so. This theme was rated second highest overall (3.98), indicating that participants think that community knowledge is important to successful report-back. The cluster had low average bridging (0.29) relative to the mean, indicating that this is a cohesive theme. The issues most salient to this theme were those addressing the challenges around community-academic dynamics: “undervaluing community knowledge”, “assumption that community doesn’t understand”, “cognitive dissonance between researchers and community”, and “bias against community members from academics” (Table 2, Statements 20, 9, 18, 14). While being the most representative of the community knowledge theme, these issues were also rated the lowest, perhaps due to the wording of the rating question which asked how “important” an issue was to successful report-back. Challenges in engaging communities may not have been considered an important strategy for researchers to employ when reporting back, but their emergence as a cohesive subtheme indicates that they are a specific issue relevant to reporting back. Conversely, the issues that participants viewed as most important to successful report-back were positively oriented and related to ways that researchers can incorporate community knowledge: “including community input on report-back process”, “ensuring community concerns are reflected in the report-back”, and “using cultural competence” (Table 2, Statements 3, 4, 26). Participants saw these issues, as related to “Effective Communication Strategies”, demonstrated by their higher bridging values. Some participants also viewed certain community issues as related to institutional review issues (“results may not be satisfactory to the community”, Figure 2 and Table 2, Statement 33) and with communication issues (“being able to reach people for report-back”, Figure 2 and Table 2, Statement 16), as demonstrated by the high bridging between these two issues and these other themes (Figure 2).

#### 3.2.3. Cluster Three: Uncertainty

The issues identified within this theme reflected factors researchers face related to the concept of uncertainty when communicating results to participants, highlighting several struggles that researchers may confront when developing and sharing report-back materials, or even deciding whether to report back at all. This theme had moderate average rating (3.85) and bridging (0.38), with many participants viewing several issues in this theme as related to others (See Figure 2). The issues that were both salient to this theme and viewed as very important to report-back reflected practical issues researchers face when developing the content of report-back materials (“deciding what to report” and “being able to talk about uncertainty,” Table 2, Statements 12 and 15). Another conceptually consistent (i.e., low bridging) issue participants identified pertaining to uncertainty was “outlining what factors/sources are contributing to the results”, (Table 2, Statement 31), and was also not viewed as very important to successful report-back, as demonstrated by its low rating.

Among the report-back issues related to uncertainty, participants thought that “not having a standard for comparison” and “differentiating between research results and diagnosis (sub-clinical results)” (Table 2, Statements 13 and 24) were the least important to successful report-back. They also saw these issues as highly related to Effective Communication Strategies and Empowering Action (Figure 2).

#### 3.2.4. Cluster Four: Empowering Action

This theme reflects the desire for successful report-back to motivate or empower action from the community. The issues in this theme highlight diverse constituents or audiences, including clinical providers, as well as environmental justice communities, and was rated fourth (3.77) out of the five themes. This theme had relatively high average bridging (0.4), indicating that many participants thought these issues were related to others. By a notable margin, the most highly rated issue referenced community resources (“The ability to act given socio-economic disparities”), and was also an anchor within this theme, indicated by its low bridging value (Table 2, Statement 30). The next three most important issues referred to providing information that is useable and actionable (“what kind of recommendations can we make”, “what do they do with [the information]”, and “can the information be used to solve the problem,” Table 2, Statements 29, 10, 25). The issue of whether “the information [can] be used to solve the problem” (Table 2, Statement 25) was also very pertinent to this theme, having the lowest bridging value. The two issues identified as the least important referenced the medical community (“How to include clinical recommendations when appropriate” and “engaging medical care providers”), and were also seen by many participants as related to issues with Institutional Review and Oversight and Uncertainty (Table 2 and Figure 2, Statements 1 and 6).

#### 3.2.5. Cluster Five: Institutional Review and Oversight

This theme reflects the logistics of receiving IRB approval to report back, as well as the challenges associated with considering the risks and benefits of reporting back. Participants viewed the issues within this theme as the least important to successful report-back, while also the most interrelated with other themes, receiving the lowest average rating (3.37) and the highest average bridging (0.62) of the five themes (Table 2). The issue within this theme seen as the most important to successful report-back was “Getting IRB approval to do report-back” (Table 2, Statement 32). Other issues within this theme reflect the challenges associated with considering the risks and benefits of reporting back (“Concerns about telling them what to do/what not to do”, “Unanticipated negative consequences beyond consented individual”, and “Tension in scientific community around right to know vs. not doing harm,” Table 2, Statements 22, 21, 2). Participants also identified the “Composition of the IRB (community representative)” as an issue relevant to reporting back (Table 2, Statement 17). Of all of the issues identified by participants, “Managing media” (Table 2, Statement 23) was seen as least important to successful report-back.

Again, this cluster had the highest average bridging of all clusters, reflecting how much this cluster interrelates with other report-back themes. The three issues with the highest bridging values were seen as relating to issues with Empowering Action and Community Knowledge and Concerns (Figure 2).

## 4. Discussion

### 4.1. Overview

**Figure 3:**
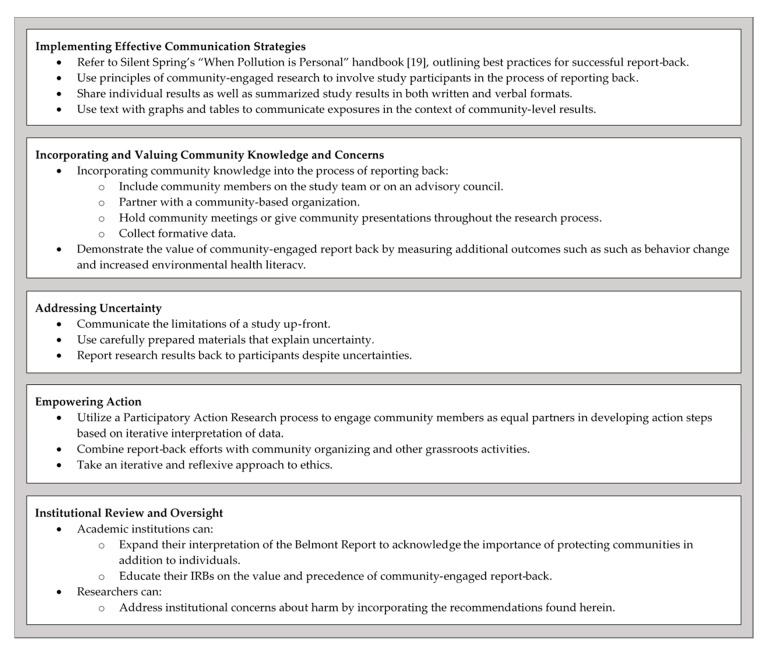
Recommendations to Improve the Practice of Reporting Back Research Results. To improve the practice of reporting back, researchers and academic institutions may consider taking action in these five areas.

The perspectives of the PEPH community about reporting back environmental health research results were identified and visually depicted using concept mapping. Through this process, five themes emerged from many inter-related issues. These five themes reflect the goals of reporting back environmental health research results, the essential elements of effectively reporting back, the challenges, and the balance between beneficence and justice. PEPH community members identified effective communication as the most important concept to consider when reporting back environmental health research results. The concept map (Figure 1), which serves as a visual framework, depicts two nearby themes also identified as highly important to reporting back: Community Knowledge and Concerns and Uncertainty. This relational spacing suggests that our workshop participants consider the integration of community knowledge in the report-back process as an essential component to the effective communication of environmental health research results. Similarly, the close proximity of the Uncertainty theme reflects the challenge of effectively communicating the uncertainties that often exist in the context of environmental health research results. As shown visually on the concept map, the Empowering Action theme and Institutional Review and Oversight themes are slightly separated from the three higher rated themes, and while these two themes are important elements of reporting back, they are not as essential to successfully reporting back environmental health research results. In fact, empowering action, with its moderate rating, appears to be a secondary goal (or potentially a long-term goal) of successful report-back, a goal that should be considered but not necessarily required when reporting back. Furthermore, empowering action’s location on the concept map between the Institutional Review and Oversight theme and the Uncertainty theme reflects the need to assess the risks and benefits of empowering action and the challenge of doing so when uncertainties exist. Lastly, the proximity of the Institutional Review and Oversight theme to the Community Knowledge and Concerns theme reflects a mechanism for improving the review process to better assess the potential impact of reporting back on individuals and on communities. In accordance with grounded theory and qualitative content analysis [15,17,18,26], we interpret the distinct themes, their importance, how they relate to each other within a conceptual framework, and, building from this framework, make recommendations to improve the process of reporting back environmental health data (summarized in Figure 3).

### 4.2. Discussion by Theme

#### 4.2.1. Effective Communication Strategies

Receiving the highest rating among themes, our workshop participants viewed effective communication as an essential element of successfully reporting back research results. The challenges noted by participants suggest that they are most concerned with the practical considerations involved in the process of designing strategies for effective science and risk communication with participants in research studies. These challenges, which include determining what message(s) to deliver, what methods to use to communicate these messages, and how and when to best deliver these messages to their intended audience, have also been documented in the report-back literature as central to designing effective communications strategies [3,7,9,27,28].

The PEPH concept mapping exercise revealed that our participants are also concerned with communicating uncertainty, as reflected by the close proximity, or interrelationships, between these two themes (Figure 2). Careful messaging is certainly important to ensure researchers neither overstate nor understate potential risks, especially when there is not sufficient research tied to health effects of emerging contaminants [27]. In addition, the relationship between “medical and environmental health literacy” and Uncertainty (Table 2 and Figure 2, Statement 8) illustrates the importance of taking the environmental health literacy of the audience into account when communicating uncertainty, a potentially difficult concept for communities or individuals to grasp in the context of receiving results.

Indeed, as the concept map created by our participants visualizes, effective communications strategies are closely related to community knowledge and concerns and uncertainty (Figure 2). Engaging community members can help researchers determine what information to deliver to study participants (e.g., appropriate level of concern, definitions of terms; Table 2, Statements 11 and 27) and how to deliver it (e.g., what medium to use, visual representations, languages, etc.; Table 2, Statements 7, 19, 28). When researchers engage their participants/community from the beginning in understanding the realistic boundaries of the study, the types of results that may be available at the individual and/or community level, and the uncertainties inherent in science, community members can guide researchers in determining what information to share and how to best deliver it to “make sure it is understandable” [3,28]. Community members perceive risk through their own personal context—and engaging them throughout the research process can build trust and allow participants to manage risk more effectively when presented with uncertainty [3,29]. Community members can also advise on which language(s) to use, communications media and outlets to employ, and provide feedback on visual representations of results, to ensure that report-back communications are understood and address any uncertainty that exists [3,7,28].

##### Recommendations

To guide research teams engaged in sharing personal exposure results, Dunagan et al. [7] developed a handbook outlining best practices for reporting back for researchers to consider, as they develop their communications approaches. One of the practices included in this handbook is to involve study participants in the report-back process. In fact, several concerns that our PEPH participants shared regarding reporting back may be addressed by using principles of community-engaged research, in which researchers work alongside community members, allowing them to “know their audience,” a key tenet of any communications effort. Community partners may better understand how study participants within a community with varied levels of literacy and numeracy process information, which is important to consider for effective communication of environmental health research results. Another recommended practice is to share individual results, as well as summarized study results, in both written and verbal formats. Using text with graphs and tables to communicate exposures in the context of community-level results has been used effectively in several different studies, especially when paired with in-person conversations about results between researchers and individuals or groups [7,9,27,28].

#### 4.2.2. Community Knowledge and Concerns

Participants in our concept mapping exercise felt that community knowledge was an essential element of successfully reporting back research results, but also identified barriers to including community knowledge. Lessons from community-based participatory research (CBPR), which emphasizes the participation of non-academic researchers in the creation of knowledge [30], may be applied to the report-back process to better incorporate community knowledge, while also addressing its challenges. CBPR principles are relevant to reporting environmental health research results in that they foster co-learning, which helps ensure that results are disseminated and intervention strategies are developed that reach more community members and are useful, relevant, and culturally appropriate [31,32,33,34].

Despite CBPR’s well-documented benefits, our participants identified academic and community dynamics that present barriers to involving the community (Table 2, Statements 9, 14, 18, 20, 33). Contributors to these barriers in academic systems likely include a lack of formal training in community engagement [35], tenure and funding requirements that do not value the practice [35], and traditional research paradigms that emphasize objective knowledge that is free of context and does not value subjective and experiential knowledge [30]. Specific to reporting back biomonitoring results, in traditional research paradigms, scientists and medical experts unilaterally make decisions about how and what to report back, while the CBPR approach involves equal participation by researchers and participants and incorporates community knowledge into report-back decisions [1]. Community dynamics, particularly individuals’ broader context, is particularly important to reporting back environmental exposure data because it influences a person’s “exposure experience”, their expectations, and how they will respond to the results [3]. Context and community knowledge are also relevant to other issues raised by our participants. For example, the institutional review process (Table 2, Cluster 5), which is grounded in the traditional research paradigm, often clashes with the goals and practices of community-engaged research and reporting back environmental exposure results [36]. This may explain some of the interconnections (i.e., bridging) between these themes (see Figure 2).

##### Recommendations

Incorporating community knowledge into the process of reporting back involves shifts by both institutions and individual researchers. As a first step in shifting academic paradigms to value co-learning and understand community contexts, academic institutions should implement CBPR trainings [35]. Individual researchers can help demonstrate the value of community-engaged report-back by measuring outcomes such as behavior change, increased health awareness, improved rigor, relevance and reach of science, increased community cohesion and engagement, individual and community action, and increased environmental health literacy [28,31,37,38]. Researchers can incorporate community knowledge into their report-back process by including community members on the study team [32,33] or on an advisory council [32,39], partnering with a community-based organization [32,40], holding community meetings or giving community presentations throughout the research process [3,32,40], and collecting formative data [40].

#### 4.2.3. Uncertainty

Participants in the concept mapping session shared concerns over issues related to uncertainty when reporting results back to study participants. Uncertainty in environmental health research can take many forms—it may relate to findings of emerging contaminants where potential outcomes are unknown, or to undiscovered sources of contamination that make it difficult to take health-protective actions. In particular, participants shared concerns about “being able to talk about uncertainty” (Table 2, Statement 15), which may relate to the ability of researchers to effectively communicate risk, especially in light of scientific uncertainty where health-protective actions may be unclear [36,41]. This interrelationship between effective communication and uncertainty is reflected in the bridging and proximity between these clusters in the concept map (see Figure 2). Ultimately, whether it is regarding unknown health effects, lack of state or federal guidelines, or understanding how best to take health protective actions—communicating about scientific uncertainty, especially to a concerned community, is one of the thorny issues facing environmental health researchers, and one that certainly influences their decision over what to report to study participants, if anything [41].

##### Recommendations

Researchers have found that participants with varying literacy and diverse backgrounds are able to understand and feel some control over their situation when presented with carefully prepared materials that explain the uncertainty [41,42]. Communicating the limitations of a study upfront (i.e., “this is a study of exposure to certain contaminants, but will not help us understand its relation to specific health outcomes”) can be particularly important when reporting back results in the face of uncertainty, though it will likely remain a challenging request by study participants who want to understand their personal risk [28]. Concerns over whether and how to discuss uncertainty in reporting back environmental health research results should not prevent researchers from reporting back. There is evidence that effective report-back can increase awareness of the effects of environmental exposures, as well as environmental health literacy in general, even when researchers may be unable to fully resolve uncertainties inherent in the results [28,32,37,43].

#### 4.2.4. Empowering Action

Empowering action was rated second to last, indicating participants see this as a secondary goal of reporting back and/or relatively less essential than other clusters to ensure successful report-back. The lower priority given to the category, coupled with the diverse audiences identified, suggests that our participants do not yet see reporting back as an effective, or perhaps appropriate, method to enable action in the communities with whom they engage. Instead, the workshop participants may view report-back primarily as a tool for enhancing community knowledge, sharing data, and clarifying the scientific process, as shown in the literature more broadly [44,45].

“The ability to act given socio-economic disparities,” (Table 2, Statement 30) was seen as the most important issue related to empowering action, recognizing that, even when armed with good data, some communities may lack the resources to enact change in their area. Others who work in community engagement and public health have shown that reporting of data alone is insufficient to enact change in environmental justice communities [46]. Conversely, issues referencing medical providers and clinical recommendations were seen as the least important issues related to empowering action (Table 2, Statements 1 and 6), showing that, while the medical community is a potential partner or audience, they are not necessarily the primary audience when reporting back. These issues were related to Institutional Review and Oversight and Uncertainty (Figure 2), reflecting the realities of institutional relationships that may affect IRB approvals when clinical recommendations are being proposed, the tension between the potential risks and benefits of empowering action, as well as the challenge of doing so in the context of uncertainty.

Other issues related to empowering action identify gaps in the process of research to action: “what kind of recommendations can we make”, “what can they do with it”, and “can the information be used to solve the problem” (Table 2, Statements 29, 10, 25). These issues of research to action were most pertinent to the concept of Empowering Action, and illuminate the challenges faced by researchers as they attempt to define what specific recommendations for action can be made.

##### Recommendations

Much of the research focusing on empowering action related to environmental concerns addresses environmental management strategies for conservation [47,48,49,50] or influencing individuals to engage in more environmentally friendly behaviors [51,52,53]. These outcomes are less salient for individuals who are responding to their own personal or community-level data. A participatory action research process, which engages community members as equal partners throughout the research process and is structured to develop action steps based on iterative interpretation of data [54,55], may be one strategy to fill this gap and make empowering action more central to the report-back process [37]. Additionally, employing a communications strategy that includes several different approaches (e.g., simple graphics along with text) may be most effective at helping participants understand their results well enough to feel empowered to take action [9]. Importantly, data sharing should be combined with community organizing and other grassroots activities [46], as well as an iterative and reflexive approach to ethics [56] to be effective.

#### 4.2.5. Institutional Review and Oversight

This theme was seen as the least important to successfully reporting back research results, and as highly interrelated with the other themes, suggesting that institutional review and the consideration of the risks and benefits of reporting back may be both influenced by other elements essential to reporting back and may also impact these other essential elements. While this theme was not as cohesive as others, it is notable that the most important issue within this theme was also the issue most interconnected with other themes, “getting IRB approval for providing report-back” (Table 2, Statement 32), which reflects the practical and sometimes challenging requirement of needing IRB approval to report back. It is possible that the simultaneously high importance and interconnectedness of this issue reflects how many of the challenges from other themes must also be considered in the institutional review process. The emphasis on the need to consider impacts of reporting back beyond the individual participant is also significant to this theme (“Unanticipated negative consequences beyond consented individual”, Table 2, Statement 21). Interestingly, this theme included a specific strategy to help better assess the risks and benefits of reporting back: including a community representative on the IRB (“Composition of the IRB (community representative)”, Table 2, Statement 17), presumably as a strategy to facilitate academic institutional review board’s awareness of the perspectives and needs of the individuals and communities they serve to protect.

Ethical concerns are in the forefront of the numerous IRB discussions about reporting back. Interpretations of the beneficence principle of human subjects research as defined by the Belmont Report [57] were invoked to oppose reporting individual results back to communities [58,59]. Efforts to report back research results were especially opposed if the results included emerging contaminants [36]. Ohayon and colleagues [41] suggested that IRB members had concerns about the potential harm to participants, such as anxiety when receiving back their own chemical exposure results, and noted the possibilities of counterproductive behavior changes. As a result of this discussion process with members of IRBs, many researchers have focused on how to develop recommendations and support for study participants that improved positive action associated with their report-back information [60], which may explain the relationship between this and the Empowering Action theme (Figure 2). In the context of the right-to-know principle [1], individuals have the right to know the chemicals to which they may be exposed in their daily living, and as such, environmental health researchers have the responsibility to inform individuals of these exposures [61].

##### Recommendations

To address institutional review issues affecting report back, academic institutions should expand their interpretation of the Belmont Report, acknowledging the importance of protecting communities in addition to individuals [36]. Academic institutions should educate their IRBs on the value and precedence of community-engaged report-back, such as the findings that reporting back research results can help communities and participants cope with uncertainty, feel a sense of control, and take health protective actions [3,28,36], as well as the recent recommendation from the National Academies of Sciences, Engineering, and Medicine [4]. To help address institutional concerns about harm, researchers can incorporate recommendations found within the themes of Effective Communication Strategies, Uncertainty, and Empowering Action, especially the application of participatory action research principles.

### 4.3. Limitations

The themes and their relationships depicted from this concept mapping process were identified by a small group of community engagement practitioners representing community organizations, academic institutions, and local and federal government agencies from across the country who voluntarily chose to participate in a workshop about reporting back research results. While more participants in our workshop represented community organizations than the meeting attendees in general, the majority still represented academic institutions. Likewise, we did not require previous experience with reporting back to participate in the workshop. Despite the small number of self-selected participants, the majority of whom represented academia, all participants were practitioners and stakeholders in community-engaged environmental health research, in which reporting back is relevant, and their attendance at this annual meeting demonstrates an interest in learning about and improving report-back practice. To further disentangle, interpret, and ultimately address this complex issue, we recommend either repeating this approach, or using these results, to guide discussion with a broader audience, including more diverse perspectives, such as those of community members and institutional review staff.

## 5. Conclusions

In accordance with our combined theoretical approach of grounded theory and qualitative content analysis, concept mapping enabled us to build from the perspectives and experiences of the PEPH community to conceptualize themes and begin disentangling the complexity of issues affecting how and whether environmental research results are reported back. By identifying themes and visually depicting the spatial relationship among them, we were able to prioritize strategies identified by community engagement practitioners that contribute to more effective report-back, and importantly, make recommendations to address the challenges within and across themes. Despite strong recommendations to return research results to participants by national and international agencies, results continue to be reported back infrequently and inconsistently. This concept map can be used as a framework for planning and action to effectively report back research results. Researchers and practitioners should address these specific themes when planning their report-back activities. Likewise, future professional development opportunities could include training on these themes, for which our results can serve as a guide. Notably, engaging community partners in the process of reporting back emerged as a unifying global theme. Applying community engagement best practices could improve how researchers report back research results by understanding the community context to develop effective communication methods that address uncertainty and identify the community’s ability to act on the results, thereby addressing institutional concerns about beneficence and justice.

## Figures and Tables

**Table 1:** Concept Mapping Steps and their Application with the Partnerships for Environmental Public Health (PEPH) Community.

Steps	Step Components	2018 PEPH Workshop
Step 1 Preparation	Selecting Participants Developing Focus Prompts: Focus for Brainstorming Focus for Rating	All 2018 PEPH Annual Meeting attendees invited to participate. Brainstorming and Rating prompts developed by HERCULES staff in consultation with NIEHS PEPH staff.
Step 2 Generation of Statements	Brainstorming	Workshop participants collectively brainstormed.
Step 3 Structuring of Statements	Sorting Statements Rating Statements	Workshop participants independently sorted and rated online during workshop
Step 4 Representation of Statements	Creation of Maps	HERCULES staff used Group Concept Mapping software to create maps during workshop
Step 5 Interpretation of Maps	Statement List Cluster List Naming the Clusters Point Map Cluster Map Cluster Rating Map	Workshop participants selected ideal cluster solution during workshop, gave input on cluster labels. All workshop participants invited to participate in survey and conference calls to select final scenario and further interpret maps.
Step 6 Utilization of Maps	For Planning (e.g., action plans, needs assessment) For Evaluation (e.g., measurement, outcome assessment)	Subset of workshop participants developed summaries of results and recommendations to improve report-back among the environmental health community, reported here.

Adapted from “An introduction to concept mapping for planning and evaluation” [23].

**Table 2:** Concept Mapping Clusters, Statements, Rating, and Bridging.

Statement Number	Statement	Average Rating ^a^	Bridging Values
**Cluster One:**	**Effective Communication Strategies**	**4.24**	**0.13**
5	Making sure the information is understandable	4.95	0.1
28	What language to deliver it in	4.55	0.08
27	Communicating the appropriate level of concern	4.35	0.27
19	What medium to use/how to deliver it	4.2	0.08
11	Defining scientific measurement/terms	4	0.05
7	How to represent it visually	3.95	0
8	Medical and environmental health literacy	3.65	0.33
**Cluster Two:**	**Community Knowledge and Concerns**	**3.98**	**0.29**
3	Including community input on report-back process	4.86	0.45
4	Ensuring community concerns are reflected in the report-back	4.76	0.42
26	Using cultural competence	4.6	0.41
16	Being able to reach people for report-back	4.4	0.64
20	Undervaluing community knowledge	3.6	0.04
14	Bias against community members from academics	3.55	0.11
18	Cognitive dissonance between researchers and community	3.4	0.1
33	Results may not be satisfactory to the community	3.4	0.39
9	Assumption that community doesn’t understand	3.2	0.05
**Cluster Three:**	**Uncertainty**	**3.85**	**0.38**
12	Deciding what to report	4.3	0.28
15	Being able to talk about uncertainty	3.9	0.25
31	Outlining what factors/sources are contributing to the results	3.75	0.33
24	Differentiating between research results and diagnosis (sub-clinical results)	3.74	0.55
13	Not having a standard for comparison	3.55	0.46
***Cluster Four:***	***Empowering Action***	***3.77***	***0.4***
30	The ability to act given socio-economic disparities	4.25	0.3
29	What kind of recommendations can we make	4.15	0.26
10	What do they do with it	4	0.3
25	Can the information be used to solve the problem	3.9	0.21
1	How to include clinical recommendations when appropriate	3.29	0.45
6	Engaging medical care providers	3.05	0.89
**Cluster Five:**	**Institutional Review and Oversight**	**3.37**	**0.62**
32	Getting IRB approval to do report-back	4.2	1
22	Concerns about telling them what to do/what not to do	3.7	0.33
17	Composition of the IRB (community representative)	3.4	0.79
21	Unanticipated negative consequences beyond consented individual	3.2	0.42
2	Tension in scientific community around right to know vs. not doing harm	2.9	0.51
23	Managing media	2.85	0.67

^a^ Rating on a scale of 1–5, with 5 being the highest.
